# The common bipolar phenotype in young people

**DOI:** 10.1186/2194-7511-1-19

**Published:** 2013-10-01

**Authors:** Philippa L Rock, Rebecca A Chandler, Catherine J Harmer, Robert D Rogers, Guy M Goodwin

**Affiliations:** Department of Psychiatry, University of Oxford, Oxford, OX3 7JX UK; Cambridge Cognition, Tunbridge Court, Tunbridge Lane, Bottisham, Cambridge CB25 9TU UK; School of Psychology, Bangor University, Bangor, LL57 2AS UK

**Keywords:** Bipolar disorder, Co-morbidity, Affective disorders

## Abstract

**Background:**

Mood elevation is common in adolescents and young adults. The aim of this study was to investigate the prevalence of a bipolar diagnosis and co-morbidity in individuals identified by online screening for experience of (hypo)manic symptoms in order to better define the common bipolar phenotype in young people.

**Methods:**

Survey data regarding experience of (hypo)manic symptoms and occurrence of co-morbidities were analysed for 106 students satisfying criteria for probable bipolar syndrome (≥7 mood elevation symptoms plus problems on the Mood Disorder Questionnaire (MDQ)), 459 threshold bipolar students (≥7 symptoms only) and 637 controls (zero symptoms). Co-morbidities investigated included neuroticism, depression, substance misuse, gambling, health problems and medication use. Twenty-one students satisfying criteria for probable bipolar syndrome, 71 threshold bipolar students and 43 controls were interviewed with the Mini International Neuropsychiatric Interview-Plus for diagnoses of DSM-IV-TR bipolar disorder I, II or not otherwise specified (NOS).

**Results and discussion:**

There was a higher incidence of bipolar diagnosis in probable bipolar (62%) compared to threshold bipolar (34%) individuals. The probable bipolar group had increased risk of co-morbidity for neuroticism, depression, substance misuse, gambling, health problems and medication use, shared to a lesser extent by the threshold bipolar group. Self-report screening for mood elevation detects a bipolar phenotype common in young people. It provides a bridge to bipolar disorder of potential interest in understanding psychopathology, treatment and prevention.

## Background

Bipolar disorder is a serious and recurrent disease that is one of the leading causes of worldwide disability (Murray and Lopez 1997). The lifetime prevalence of bipolar disorder is around 4% to 5% (Merikangas et al. 2007) if hypomania and other minor elated states are included in its definition (Akiskal et al. 2000). Indeed, mood elevation is the core and defining psychopathology of bipolar disorder. When severe (mania), it is impairing and a problem in itself. When milder (hypomania or sub-syndromal), it essentially modifies the major depression diagnosis, with which patients may present, or contributes to mood instability. Mood elevation is therefore a phenomenon which has the potential to bridge between DSM-5 categorical diagnoses and the Research Domain Criteria project (RDoC) launched by NIMH (Insel et al. 2010). The latter refers to 'dimensions of observable behavior and neurobiological measures’, and clearly, mood elevation is a key construct, which relates to positive valence systems identified in the initial RDoC matrix. The problem remains however that patients presenting for care will define the population of interest but will be difficult to study free of medications and of the burdens of cognitive impairment and psychological/physical co-morbidity typical of mature patient samples. One solution, hitherto surprisingly neglected, may be to study symptom dimensions in non-clinical populations.

Research in adolescents and young adults has indicated that hypomanic experience is a common adolescent phenomenon in the general population (Tijssen et al. 2010a). Even rates of DSM-IV diagnosed disorder are higher than would be expected, but repeated sampling for mood symptoms in young people shows that outcomes may be benign (Lewinsohn et al. 2000; Tijssen et al. 2010b). However, even if hypomanic experience during adolescence is infrequently associated with conversion to bipolar disorder (Tijssen et al. 2010a), it is of potential interest for understanding the psychopathology of mood elevation per se and particularly any associated co-morbidity (Lewinsohn et al. 2003,2004). Co-morbidity is a major feature of established bipolar disorder, which greatly contributes to problems in management.

There has been little work to establish the validity of a continuum approach in psychological or neurobiological studies that would include sub-syndromal experience as well as categorical bipolar disorder. This is despite the fact that 'bipolar disorder’ defined by clinical interview in a young population not seeking help for their psychiatric symptoms may be regarded more appropriately as simply the more severe end of the risk continuum rather than an established lifetime condition (Beesdo et al. 2009).

The current study investigated undergraduate students who had completed an online screening questionnaire assessing psychological health. Its objective was to use a simple, cheap method of access to young people to better define the syndromal and sub-syndromal experience of mood elevation identified by self-report and to relate this to risk factors and co-morbidities previously described in patient samples. It demonstrates the feasibility of online assessment and recruitment and confirms the key similarities (and differences) between syndromal and sub-syndromal hypomanic psychopathology, which is common in young people.

## Methods

### Participants

Undergraduate students voluntarily completed an online screening questionnaire assessing psychological health as part of the University of Oxford Student Stress Survey. Survey data were collected over five consecutive years (2004–2009) from 2,591 students using an online link accessed from an email that explained the study and invited their participation. This research was approved by the University of Oxford Central University Research Ethics Committee.

The online questions collected a range of demographic and personal information. The 12-item General Health Questionnaire (GHQ) (Goldberg and Williams 1988) provided an indication of current psychological distress. GHQ responses were scored using a binary system (0-0-1-1; maximum score = 12); a cut-off of three items was used for GHQ-12 'caseness’ (Goldberg and Williams 1988). When scores on the GHQ exceeded this cut-off, the nine-item Patient Health Questionnaire (PHQ) was used to probe for current depressive episodes (threshold score = 15; maximum score = 27) (Kroenke et al. 2001). Previous history of depression was also assessed. Eysenck's Personality Questionnaire (EPQ) (Eysenck et al. 1985) was included as a measure of neuroticism (maximum score = 15). The CAGE questionnaire (Mayfield et al. 1974) for alcohol dependency (threshold score = 2; maximum score = 4) and questions about gambling frequency and online gambling were included. Simple questions about current and past health problems and use of medications were also included.

The comparison groups were selected based on responses about experience of mood elevation symptoms using questions modified from the Mood Disorder Questionnaire (Hirschfeld et al. 2000); this is a self-report screening tool to identify the symptoms that contribute to a DSM-IV-TR diagnosis of (hypo)mania. Criteria for the 'probable bipolar syndrome’ required experience of at least 7 of the 13 mood elevation symptoms and both endorsement of the co-occurrence and problematic nature of the symptoms (Hirschfeld et al. 2000). We defined a 'threshold bipolar syndrome’ as at least seven mood elevation symptoms but without both co-occurring and problematic symptoms. Control individuals were selected who endorsed no mood elevation symptoms at all.

### Psychiatric interview

Twenty-one individuals who satisfied criteria for the probable bipolar syndrome, 71 individuals who satisfied criteria for the threshold bipolar syndrome and 43 asymptomatic controls agreed to attend for face-to-face diagnostic interview. These individuals had indicated that they were happy to be contacted for further research and were simply the first able to attend for interview. The Mini International Neuropsychiatric Interview-Plus (MINI-Plus) (Sheehan et al. 1998) was used to screen these participants for DSM-IV-TR diagnoses of bipolar I, II or not otherwise specified (NOS). The positive predictive value of the MDQ for bipolar diagnosis was calculated for probable bipolar syndrome and threshold bipolar syndrome groups as True positives/(True positives + False positives). This research was approved by the Oxfordshire Research Ethics Committee.

### Statistical analyses

All data were analysed with SPSS statistical software (version 19.0 for Mac; IBM Corp., Armonk, NY, USA) to test for differences between students in the probable bipolar, threshold bipolar, and zero-symptoms groups. Continuous data were analysed using one-way ANOVAs (two-tailed) to test for differences between groups, and *η*^2^ effect sizes were calculated. Where relevant, in the case of a significant main effect of group, post-hoc *t* tests were carried out. Dichotomous data were analysed with likelihood ratio chi-square tests, for which *ϕ* effect sizes were calculated. A significance threshold of *p* = 0.05 was used for all analyses.

## Results

### Survey data

Of the 2,591 participants in the database, 106 (4.1%) (46 males, 60 females) satisfied the criteria for probable bipolar syndrome (at least seven mood elevation symptoms with concurrent symptoms causing moderate-severe problems). A further 459 (17.7%) (216 males, 243 females) satisfied the threshold bipolar criteria and had experienced at least seven mood elevation symptoms (without endorsing both the co-occurrence and problematic nature of symptoms), and 637 (24.6%) (282 males, 355 females) provided the control group with no mood elevation symptoms at all. The three groups were well matched for age (probable bipolar 19.4 ± 3.9 years vs. threshold bipolar 19.0 ± 1.8 years vs. controls 19.0 ± 1.3 years) and gender.

Prevalences of different symptoms of mood elevation are compared for the probable bipolar and threshold bipolar groups in Table 1. Behavioural symptoms of mood elevation were particularly frequently endorsed by individuals with the probable bipolar syndrome. There was a more than 20% difference in frequency for items capturing hyper behaviour, risk-taking behaviour, irritability and irresponsible spending. By contrast, core subjective symptoms of mood elevation were endorsed almost equally often in the probable bipolar and threshold bipolar groups, with intermediate exceptions of racing thoughts and distractibility.

**Table 1:** **MDQ symptoms score and prevalence of mood elevation symptoms in survey sample**

	Probable bipolar (***N***= 106)	Threshold bipolar (***N***= 459)	Difference	Significance
MDQ symptoms score: mean (s.d.)	9.7 (1.9)	8.5 (1.5)	1.17	*F* = 48.135, *p* < 0.001; *η*^2^ = 0.079
Frequency of occurrence of individual symptoms				
Marked differences				
Hyper behaviour leading to trouble (%)	81	50	31	LR = 35.847, *p* < 0.001; *ϕ* = 0.242
Risk-taking behaviour (%)	82	58	24	LR = 23.732, *p* < 0.001; *ϕ* = 0.196
Irritability leading to shouting (%)	79	56	23	LR = 21.606, *p* < 0.001; *ϕ* = 0.189
Irresponsible spending (%)	27	7	20	LR = 30.162, *p* < 0.001; *ϕ* = 0.256
Slight differences				
Racing thoughts (%)	85	73	12	LR = 7.146, *p* = 0.008; *ϕ* = 0.108
Distractibility (%)	90	80	10	LR = 6.491, *p* = 0.011; *ϕ* = 0.101
Minor or no differences				
Increased interest in sex (%)	70	65	5	LR = 1.098, *p* = 0.295; *ϕ* = 0.044
Increased talkativeness (%)	80	75	5	LR = 1.340, *p* = 0.247; *ϕ* = 0.048
Increased sociability (%)	61	61	0	LR = 0.004, *p* = 0.952; *ϕ* = 0.003
Increased confidence (%)	84	85	-1	LR = 0.041, *p* = 0.840; *ϕ* = -0.009
Decreased need for sleep (%)	68	69	-1	LR = 0.074, *p* = 0.786; *ϕ* = -0.011
Increased energy (%)	85	88	-3	LR = 0.625, *p* = 0.429; *ϕ* = -0.034
Increased activity (%)	73	83	-10	LR = 5.095, *p* = 0.024; *ϕ* = -0.098

s.d., standard deviation; LR, likelihood ratio; *df* = 563 for MDQ symptoms score or *df* = 1 for other variables.

Questionnaire scores for neuroticism, depression, substance misuse, gambling, health problems and medication use are compared for individuals with the probable bipolar syndrome, individuals with the threshold bipolar syndrome, and controls in Table 2. In general, there is a gradient across groups in all measures, highest in the probable bipolar syndrome group and lowest in controls.

**Table 2:** **Questionnaire scores for individuals with the probable bipolar or threshold bipolar syndrome and controls**

	Probable bipolar (***N***= 106)	Threshold bipolar (***N***= 459)	Controls (***N***= 637)	Significance
EPQ-N, mean (s.d.)	9.4 (3.5)	8.1 (3.5)	5.3 (3.3)	*F* = 124.772, *p* < 0.001; *η*^2^ = 0.172
GHQ-12 caseness (≥3) (%)	77	58	47	LR = 41.807, *p* < 0.001; *ϕ* = 0.183
Current PHQ ≥ 15^a^ (%)	33	10	4	LR = 53.477, *p* < 0.001; *ϕ* = 0.279
Past PHQ ≥ 15^a^ (%)	54	21	9	LR = 88.999, *p* < 0.001; *ϕ* = 0.336
CAGE ≥ 2 (%)	34	22	6	LR = 90.137, *p* < 0.001; *ϕ* = 0.274
Gambles several times a month^b^ (%)	6	7	3	LR = 7.010, *p* = 0.030; *ϕ* = 0.105
Gambles online^b^ (%)	13	8	2	LR = 16.574, *p* < 0.001; *ϕ* = 0.165
Health problems (%)	28	14	8	LR = 31.158, *p* < 0.001; *ϕ* = 0.174
Medications^a^ (%)	6	2	0.4	LR = 13.197, *p =* 0.001; *ϕ* = 0.183

s.d., standard deviation; LR, likelihood ratio; *df* = 2, 1199 for EPQ-N or *df* = 2 for other variables. ^a^Probable bipolar group: total *N* = 80; threshold bipolar group: total *N* = 345; controls: total *N* = 499. ^b^Probable bipolar group: total *N* = 53; threshold bipolar group: total *N* = 236; controls: total *N* = 347.

Neuroticism levels assessed by the Eysenck Personality Questionnaire (EPQ-N score) were significantly affected by group, with individuals with both the probable bipolar syndrome (*t*_741_ = 11.747, *p* < 0.001) and the threshold bipolar syndrome (*t*_1,094_ = 13.349, *p* < 0.001) having substantially higher scores than controls and individuals with the probable bipolar syndrome having significantly higher neuroticism levels than those with the threshold bipolar syndrome (*t*_563_ = 3.497, *p* = 0.001).

GHQ scores were analysed using a threshold score of 3 out of 12 as an indication of current caseness. Even using this liberal definition, caseness frequency was significantly affected by group, with the highest frequency in individuals with the probable bipolar syndrome, followed by individuals with the threshold bipolar syndrome, then controls. The frequencies of current and past depressive episodes, estimated by taking PHQ scores over a threshold score of 15 (out of 27), showed a similar pattern of results.

CAGE scores for increased risk of alcohol dependency and frequency of students who gamble several times a month or who gamble online were significantly modulated by group, with the highest rates in individuals with the probable bipolar syndrome, followed by individuals with the threshold bipolar syndrome, then controls.

The frequency of occurrence of health problems and use of medications were significantly modulated by group, with the highest rates in individuals with the probable bipolar syndrome, followed by individuals with the threshold bipolar syndrome, then controls.

### Psychiatric interview

The sub-sample of participants completing the psychiatric interview included a total of 135 students (61 males, 74 females). Twenty-one students (10 males, 11 females) satisfied the full screening criteria for probable bipolar syndrome, 71 students (31 males, 40 females) satisfied the threshold bipolar criteria, and there were 43 controls (20 males, 23 females). The three groups were well matched for age (probable bipolar 19.5 ± 1.2 years vs. threshold bipolar 19.8 ± 1.3 years vs. controls 20.2 ± 1.4 years) and gender.

The results of the psychiatric interview with the MINI-Plus are shown in Table 3. These interviews revealed a generally good agreement between interview and questionnaire estimates of bipolar experience. Thus, there were no bipolar diagnoses in the zero-symptoms control group. A score of 7 on the MDQ, as expected, predicted an increased rate of DSM-IV-TR bipolar diagnosis (bipolar I, II, NOS) at interview. The positive predictive value of the MDQ for bipolar diagnosis was 62% for individuals with the probable bipolar syndrome and 34% for individuals with the threshold bipolar syndrome.

**Table 3:** **Prevalence of DSM-IV-TR bipolar diagnosis, co-morbidities, and family history of mood disorder**

	Probable bipolar (***N***= 21)	Threshold bipolar (***N***= 71)	Controls (***N***= 43)	Significance
DSM-IV-TR bipolar I, II, NOS (%)	62	34	0	LR = 39.812, *p* < 0.001
DSM-IV-TR bipolar I (%)	5	0	0	LR = 3.762, *p* = 0.152
DSM-IV-TR bipolar II (%)	38	17	0	LR = 20.837, *p* < 0.001
DSM-IV-TR bipolar NOS (%)	19	17	0	LR = 13.306, *p* = 0.001
DSM-IV-TR unipolar depression (%)	5	7	7	LR = 0.158, *p* = 0.924
DSM-IV anxiety disorder (%)	33	13	0	LR = 17.551, *p* < 0.001
DSM-IV bulimia nervosa (%)	5	3	0	LR = 2.511, *p* = 0.285
DSM-IV alcohol dependence/abuse (%)	5	10	2	LR = 2.871, *p =* 0.238
DSM-IV substance dependence/abuse (%)	10	1	0	LR = 5.053, *p* = 0.080
Family history of mood disorder^a^ (%)	75	17	10	LR = 30.164, *p* < 0.001
Family history of bipolar disorder^a^ (%)	20	1	0	LR = 11.637, *p* = 0.003

Anxiety disorders include generalised anxiety disorder, specific phobia, obsessive-compulsive disorder, social phobia, agoraphobia and panic disorder. LR, likelihood ratio; *df* = 2 for all chi-square analyses. ^a^Probable bipolar group: total *N* = 20; threshold bipolar group: total *N* = 65; controls: total *N* = 40.

On the basis of these positive predictive values and the prevalence of individuals with the probable bipolar syndrome and the threshold bipolar syndrome in the survey (4.1% and 17.7%, respectively), the extrapolated likely rate of bipolar (I, II, NOS) diagnosis in the survey sample is estimated to be at least 8.5%.

## Discussion

Our data support the existence of a common bipolar phenotype in late adolescence that can be readily identified either by interview or by online self-report. Core subjective symptoms of mood elevation (increased energy, confidence, sociability, talkativeness, interest in sex, and decreased need for sleep) were equally common in the probable bipolar and threshold bipolar groups identified by self-report. The absence of a notable increase in core symptoms in individuals with probable bipolar syndrome is consistent with a continuum of mood elevation experience in this young population. Self-reports of behavioural symptoms of mood elevation such as risk taking, irritability leading to shouting, and irresponsible spending were more common in those subjects endorsing additional items that indicate symptom-related problems, described here as probable bipolar disorder. We therefore hypothesise that failure of cognitive control is the key feature that distinguishes the two groups.

Cognitive control is a loose construct taken from contemporary cognitive neuroscience. However, its relevance to psychopathology and social behaviour could be profound. Indeed, it has been claimed that a gradient of childhood self-control predicts health, wealth and public safety (Moffitt et al. 2011), although prediction of personality disorder rather than mood disorder appears to be the main mediating psychopathology (Caspi et al. 1996). An understanding of why mood elevation leads to loss of control in some people and not in others appears to be a currently under-researched question.

Co-morbidity was commonly associated with the broad bipolar phenotype and diagnosed DSM-IV-TR bipolar disorder. This has two interesting implications. First, it offers important support to the conclusion that the phenotype may be properly regarded as bipolar. Second, it suggests that the psychopathology underlying vulnerability to anxiety, alcohol misuse and gambling is present from the beginning rather than appearing as a secondary consequence of bipolar disorder. In general, co-morbidity was elevated in both the threshold and probable bipolar groups, compared with controls not endorsing hypomanic items, but the co-morbidity was greater in the probable bipolar group. This was true for neuroticism (Eysenck et al. 1985) and scores of two items or more on the CAGE questionnaire, which is a predictor for alcohol problems (Mayfield et al. 1974).

Mood elevation was strongly associated with depressive experience assessed either as threshold scores on the General Health Questionnaire (Goldberg and Williams 1988) or more specifically with depressive episodes, assessed with the Patient Health Questionnaire (Kroenke et al. 2001). At interview, the questionnaire findings confirmed the increased rates of bipolar diagnosis in the probable bipolar and threshold bipolar groups, which were also associated with increased lifetime prevalence of anxiety disorders and the probability of a family history in the probable bipolar group.

Finally, the findings on physical illness also show a gradient of risk predicted by the severity of mood elevation. The direction of this effect and its basis are poorly understood but of considerable potential interest. It also serves to underline the validity of the (non-specific) physical co-morbidity seen in patient groups (Fenn et al. 2005).

### Limitations and strengths

There is an apparent credibility gap between the relative rarity of severe bipolar disorder in comparison with the relatively high frequency of the common phenotype described here. Even the rates of DSM-IV-TR diagnosis predicted from our interview data are high. This is not an artefact of self-report and is comparable to findings of interview-based surveys (Beesdo et al. 2009). Clearly, it implies that hypomanic experience is relatively common, probably a developmental stage for many young people rather than an illness. However, the co-morbidity with anxiety, neuroticism, depression and alcohol misuse all echo the established illness phenotype in bipolar disorder (Kessler et al. 2007). They suggest a valid cluster of psychopathological vulnerability associated with the experience of mood elevation. We have suggested before that this affords a model for studying such vulnerability unconfounded by the burden of chronic mood disorder, and with very well matched case–control samples recruited in an identical way (Rock et al. 2010; Yip et al. 2012). Moreover, it provides a starting point for RDoC-style measurements.

There is also the major challenge of how the vulnerability of the phenotype can provide a way to identify those most at risk of a later illness course. A probable bipolar diagnosis is very easy to make online and has a positive predictive value of 62% for DSM-IV-TR bipolar diagnosis (bipolar II and bipolar NOS) at interview. However, we do not know whether simply passing that threshold has important implications for later development. But again, better understanding of the mechanisms underlying mood elevation could lead to simple interventions or advice with public health implications. The desire, representing a wide consensus, to focus research and prevention earlier in the illness course (http://www.mrc.ac.uk/Utilities/Documentrecord/index.htm?d=MRC006848) necessitates grappling with currently weak predictors of later problems.

## Conclusions

In conclusion, the bipolar phenotype is an easily identified risk factor for mood disorder and is of potential interest in understanding psychopathology, treatment and prevention. The transition to more severe disorder remains poorly understood and an important target for future study.
